# Best fitting tumor growth models of the von Bertalanffy-PütterType

**DOI:** 10.1186/s12885-019-5911-y

**Published:** 2019-07-12

**Authors:** Manfred Kühleitner, Norbert Brunner, Werner-Georg Nowak, Katharina Renner-Martin, Klaus Scheicher

**Affiliations:** Institute of Mathematics, Department of Integrative Biology and Biodiversity Research, University of Natural Resources and Life Sciences (BOKU), Gregor Mendel Strasse 33, A-1180 Vienna, Austria

**Keywords:** Cancer, Simulated annealing, Tumor growth, Bertalanffy-Pütter growth models

## Abstract

**Background:**

Longitudinal studies of tumor volume have used certain named mathematical growth models. The Bertalanffy-Pütter differential equation unifies them: It uses five parameters, amongst them two exponents related to tumor metabolism and morphology. Each exponent-pair defines a unique three-parameter model of the Bertalanffy-Pütter type, and the above-mentioned named models correspond to specific exponent-pairs. Amongst these models we seek the best fitting one.

**Method:**

The best fitting model curve within the Bertalanffy-Pütter class minimizes the sum of squared errors (SSE). We investigate also near-optimal model curves; their SSE is at most a certain percentage (e.g. 1%) larger than the minimal SSE. Models with near-optimal curves are visualized by the region of their near-optimal exponent pairs. While there is barely a visible difference concerning the goodness of fit between the best fitting and the near-optimal model curves, there are differences in the prognosis, whence the near-optimal models are used to assess the uncertainty of extrapolation.

**Results:**

For data about the growth of an untreated tumor we found the best fitting growth model which reduced SSE by about 30% compared to the hitherto best fit. In order to analyze the uncertainty of prognosis, we repeated the search for the optimal and near-optimal exponent-pairs for the initial segments of the data (meaning the subset of the data for the first n days) and compared the prognosis based on these models with the actual data (i.e. the data for the remaining days). The optimal exponent-pairs and the regions of near-optimal exponent-pairs depended on how many data-points were used. Further, the regions of near-optimal exponent-pairs were larger for the first initial segments, where fewer data were used.

**Conclusion:**

While for each near optimal exponent-pair its best fitting model curve remained close to the fitted data points, the prognosis using these model curves differed widely for the remaining data, whence e.g. the best fitting model for the first 65 days of growth was not capable to inform about tumor size for the remaining 49 days. For the present data, prognosis appeared to be feasible for a time span of ten days, at most.

## Background

### Bertalanffy-Pütter differential equation

Historically, the systematic application of mathematical models for tumor growth has begun in the 1960s [1–3]. In the meantime, so many different approaches towards modeling were developed that concerns about a “model muddle” have evolved [4–6]. The focus of this paper is on longitudinal studies of tumor volume, which use tumor growth curves that are defined from certain first order ordinary differential equations [7]. Such studies aim at biophysical explanations for tumor growth and at tools for prognosis and therapy [8–10]. In this context, the Bertalanffy-Pütter [11–13] differential eq. (1) has been recommended as “a macroscopic model variant that can be conceived as an optimal condensed modeling approach that to a high degree preserves complexity with respect to … more complex modeling variants” [14]:

1 $$ \frac{dv(t)}{dt}=p.v{(t)}^a-q.v{(t)}^b $$

This equation describes tumor volume *v*(*t*) in mm^3^ over time *t* in days, using five model parameters that are to be determined from fitting the model to size-at-age data: Four parameters are displayed in the equations, namely the non-negative exponent-pair *a* < *b* and the constants *p* and *q*. A fifth parameter is the initial tumor volume at the start of monitoring, i.e. *v* (0) = *v*_0_ > 0.

**Fig. 1:**
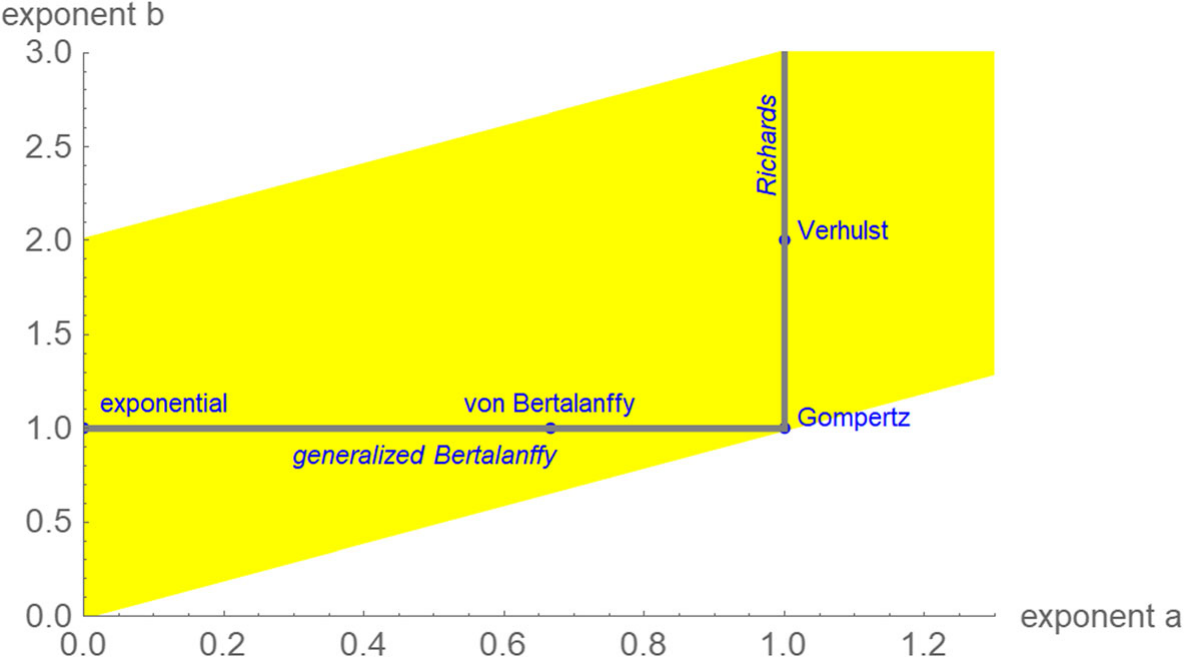
Exponent-pairs of well-known named models (blue dots and grey lines); exponent-pairs that were considered in an initial search for the best fitting model (yellow)

In this paper, we perceive eq. (1) as a definition of a two-parameter family of growth models, whereby each exponent-pair (*a*, *b*) defines a unique model with three free parameters (*p*, *q*, and *v*_0_). Thus, for these models the “model muddle” can be reduced by considering them within the context of the larger unifying class (1) of models. Figure 1 displays (in blue) several “named models” that can be defined from certain exponent-pairs and displays (in yellow) additional exponent-pairs that in view of their closeness to the named ones we deemed as biologically meaningful; we considered them for an initial search. For example, the exponent-pair (*a*, *b*) = (0, 1) defines exponential growth (i.e. *v*(*t*) = *v*_0_·e^-*q*·*t*^, assuming *p* = 0, *q* < 0), and bounded exponential growth (i.e. *v*(*t*) = (*p*/*q*)·(1-*d*·e^-*q*·*t*^), assuming *p*, *q*, *v*_0_ > 0 and defining *d* from these parameters). The logistic growth model of Verhulst [15] is defined from eq. (1) using the exponent-pair (*a*, *b*) = (1, 2). The Gompertz [16] model is the limit case *a* = *b* = 1; it uses a different differential equation [17]. These models are amongst the most common models in this field (Google Scholar: 237,000 hits for “tumor growth model, exponential growth”, 122,000 hits for “tumor growth model, logistic” and several thousand hits for other named growth models).

Richards’ [18] model (Fig. 1: grey line *a* = 1, *b* > 1) and the generalized Bertalanffy model (Fig. 1: grey line *b* = 1, 0 ≤ *a* < 1) are represented as classes of models. In the theory of economic growth, the latter model (class) is known as Solow-Swan model [19–22].

A drawback of this type of phenomenological models is the difficulty in relating the comparatively easy to observe macroscopic data (size-at-age) to actual biological processes. According to von Bertalanffy [11, 14], the parameters of eq. (1) relate to resource utilization, metabolism and morphological structures of tumors: [11] has chosen the exponent *a* = 2/3, as the inflow of energy would be proportional to the surface area (i.e. proportional to volume^2/3), and the exponent *b* = 1, as the energy needs for sustenance would be proportional to volume (cell count). This model appears to be plausible for the avascular stage of a solid tumor (nutrients enter only through the periphery). However, other authors proposed different biophysical explanations of growth and different exponent-pairs [23, 24]. Thus, the tumor surface may be fractal, whence the first exponent (*a*) may be above the value 2/3 of [11]. Further, as noted by [25], a static bio-mechanical explanation of growth may not capture growth for changing biological drivers due to e.g. the formation of new blood and lymphatic vessels (angiogenesis, lymphangiogenesis) or due to growth beyond the bounds of the original organ (extracapsular extension). [26, 27] analyzed the reasoning of [11] in the context of the biology of fish and they recommended the use of more general model classes, namely the generalized Bertalanffy model and later all models for eq. (1). Other authors recommended the analysis of the relative growth rates *v´*/*v* over time, as these would inform about metabolism [28].

A different modeling approach describes tumor growth at the more detailed tissue scale in terms of partial differential equations related to invasion-proliferation and diffusion-reaction; e.g. Fisher-Kolmogorov equation [29, 30]. For such an approach the explanations of growth rest on firm theoretical ground, but for the study of concrete tumors complex data about their spatial evolution over time would be needed; simple size-at-age data would not suffice.

### Problem of the paper

We reconsider the findings of [31]. They compared seven models. Of them, the models of von Bertalanffy, Gompertz, and Verhulst, would be “particularly popular choices for modeling tumor growth … because they include a biologically realistic slowing of the growth rate as the tumor increases. Yet it is precisely this feature that results in the poor predictive value of the models.” They supported their claim through data, where the best fitting model underestimated future tumor growth.

As these findings depended on a few models only, and as there is no generally valid tumor growth model, which ensures a clear understanding and prognosis of tumor growth, the present paper revisits this issue and considers models from a more comprehensive class. The differential eq. (1) defines such a class that encompasses the most popular models (see above). We therefore aim at comparing the models from the model class (1) in terms of their goodness of fit (see methods) to the data of [31] and we assess their utility for prognosis.

This approach has the following advantages: First, using a larger class of models with different growth patterns for comparison will provide a high flexibility in data-fitting and results in a better fit of the overall optimal model; for such a model we expect a better prognosis. Second, using a large class of models allows for a better comparability of the results with the literature. For even if a certain model may not be an element of the large class, some other element of the class may approximate it. And third, this approach allows estimating the uncertainty of prognosis. We claim that there may be limits to prognosis that are inherent to the research problem (c.f. prognosis of weather), and that near-optimal models might highlight these limits. For near-optimal models [32–34] the fit to the data does not differ significantly from the best fit model (see methods), but their forecasts may differ. This approach improves upon the traditional sensitivity analyses (variation of model parameters) insofar, as for the present models and data even slight changes in the parameters (some parameters are of size 10^− 30^) may result in very poorly fitting model curves.

This working plan has not yet been implemented in literature, because the optimization of the five parameters *a*, *b*, *p*, *q*, and *v*_0_ of (1) may fail with standard optimization software. We will therefore also explain the optimization tools.

## Methods

### Data

**Fig. 2:**
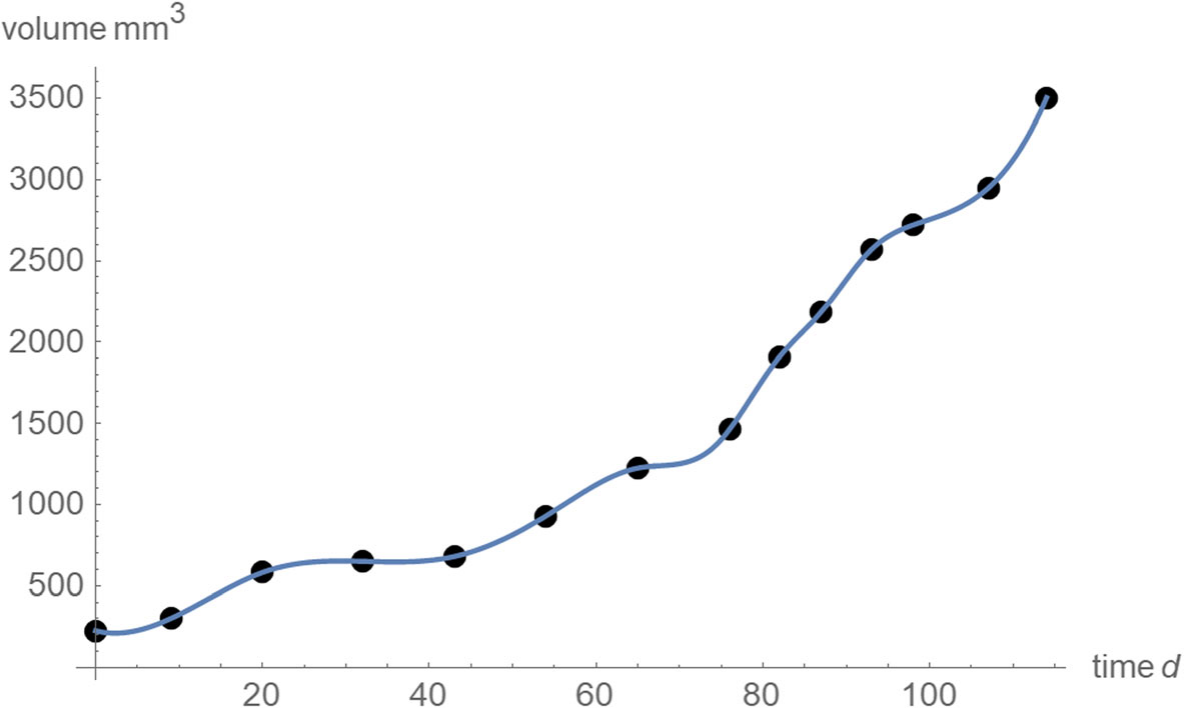
Size-at-age data (black dots) from Table 1 and cubic splines (blue). Additional statistical information (e.g. standard deviations) was not available for the original data

We aim at explaining the findings of [31] and we therefore use the data of that paper; they originate from [35]. Human breast cancer cells of type GI-101A were injected subcutaneously into nude mice and the subsequent tumor growth was observed for 114 days for a treatment group and a control group (8 animals). At 14 points of time tumor volume was measured (Table 1); Fig. 2 plots the data and an interpolation function that was obtained by the method of cubic splines [36].

**Table 1:** Size-at-age data as retrieved from a graphic

Time (days)	Volume (mm^3^)	Time (days)	Volume (mm^3^)
0	225	76	1,464
9	300	82	1,911
20	582	87	2,184
32	650	93	2,570
43	680	98	2,721
54	930	107	2,948
65	1,225	114	3,503

Note: We extracted the data from the original source, the red line in the right-hand plot of Fig. 1a of [35], using DigitizeIt® software of Bormisoft, and rounded to integers. The original data plotted the average volume *v*_*i*_ in mm^3^ at day *t*_*i*_, where *i* = 1, 2, … 14 and *t*_1_ = 0

We demonstrate our method to find the best fitting and the near-optimal models for this dataset, only, as the paper aims at a “proof of principle”. While the conclusions about the limitations of prognosis may not apply to other data, the method to obtain such conclusions for concrete data generalizes. As a visual inspection of the data (Fig. 2) would suggest that the first and the second half of the growth process may have been driven by different biological processes (steeper slope for the second half), there also arises the question, if a single model of the type of eq. (1) suffices to approximate the data accurately. (If two models were needed to describe two phases of growth, this would require 11 parameters, five for each model and one for the moment of model change. Reasonable modeling would require significantly more data-points.)

### Models

The yellow region in Fig. 1 displays the models that we considered initially; i.e. each of the displayed exponent-pairs defined a unique model of type (1) with three free parameters. For numerical purposes, we worked with an accuracy of 0.01 for the exponent-pairs, whence we considered only exponent-pairs on a grid (*a* = *m*·0.01, *b* = *a* + *n*·0.01, with integers *m* ≥ 0, *n* ≥ 1). If the exponent-pair of the best fitting model was attained on the upper boundary line or on the right boundary line of this grid (yellow region of Fig. 1), then further grid-points were added for the search to verify the optimality of this exponent-pair or replace it by a better one.

The diagonal *a* = *b* was not considered, as for exponents *a* = *b* the differential eq. (1) needs to be replaced by another differential equation from [17] of the Gompertz-type (e.g. the Gompertz-model for *a* = *b* = 1). Thus, if the exponent-pair with the best fitting model curve was a grid point next to the diagonal, then there remained the possibility that a Gompertz-type model could fit better. However, we expected that the improvement would be small. For, as noted by [17], the Gompertz-type models are limits of models of type (1) with exponents *a* < *b*.

### Fit to the data

[31] assessed the fit of models to data by means of the sum of squared errors *SSE*, which is defined as follows; *v*(*t*) is a solution of eq. (1), using certain exponents *a* < *b* and parameters *p*, *q*, *v*_0_, and *v*_*i*_ are the data from above:

2 $$ SSE={\sum}_{i=1}^n{\left({v}_i-v\left({t}_i\right)\right)}^2 $$

Thus, each exponent-pair (*a*, *b*) defines a unique model from class (1) with three free parameters (*p*, *q*, *v*_0_). For each of these models (exponent-pairs) we wished to identify the best fitting model parameters *p*, *q*, *v*_0_. Thereby, we used the method of least squares; i.e. for each exponent-pair (*a*, *b*) on the grid we sought parameters (*p*, *q*, *v*_0_) that minimized *SSE*. This defined the following function on the grid to identify, for each exponent-pair *a* < *b*, the minimal *SSE* that can be achieved:

3 $$ {SSE}_{opt}\;\left(a,b\right)=\underset{v_0,p,q}{\min }(SSE),\mathrm{assuming}\kern0.17em \mathrm{model}\;(1)\;\mathrm{with}\kern0.17em \mathrm{exponents}\;\mathrm{a},\mathrm{b} $$

In terms of this definition, an exponent-pair (*a*, *b*) from the grid of Fig. 1 (yellow region) was defined as optimal, if the function *SSE*_*opt*_ attained the minimum over the grid at this exponent-pair. The exponent-pair was defined as near-optimal, if *SSE*_*opt*_(*a*, *b*) was at most a certain percentage (we considered the thresholds 1, 5, and 10%) larger than this minimal *SSE*.

Using *SSE* to assess the goodness of fit emphasizes the accuracy of the interpolation by means of the model curve. It is also common in forecasting, whereby the Verhulst and Gompertz models are amongst the most popular growth models in that field [37, 38]. Thus, *SSE* appears to be suitable for the present paper, as the goal is the study of the limitations of the prognosis of the growth of individual tumors.

As using *SSE* is based on the implicit assumption that the fit residuals (differences between the observed data and the fitted model curve) are normally distributed, we tested this assumption using the Anderson-Darling and Cramér-von Mises distribution fit tests [39]. We investigated also an alternative statistical assumption, a lognormal distribution, where the standard deviation of volume is approximately proportional to volume. However, as the prognosis in this case tended to underestimate growth, the results are not discussed here.

Alternative criteria for model selection, e.g. the well-known Akaike information criterion *AIC* [40–42], focus on parsimony (suitable e.g. for the comparison of theories of growth). As noted by [31], for their data the most parsimonious of their models, exponential growth, falsely predicted unbounded growth. Therefore, for this paper we do not consider this approach.

### Optimization

The computation of *SSE*_*opt*_ (i.e. optimization) was done using Mathematica® 12.0 software of Wolfram Research. The authors provide a Mathematica file as supporting material. The output was exported to a spreadsheet, Microsoft Excel®. It is provided as a supporting material in the format (*a*, *b*, *v*_0_, *p*, *q*, *SSE*_*opt*_(*a*, *b*)) of the table.

For optimization, the grid-point exponent-pairs (*a*, *b*) were visited by means of an outer loop running through *a* = *m*·0.01 and an inner loop that for each *a* ran through the values *b* = *a* + *n*·0.01. Given (*a*, *b*), the optimization of *p*, *q*, and *v*_0_ was done using a custom-made variant of the method of simulated annealing [43], as this made the optimization process fully automated. A typical step of simulated annealing started with given candidates *p*, *q*, *v*_0_ > 0 for optimal parameters. The candidate parameters were altered by multiplying them with positive random numbers close to 1. (This preserved positivity in order to obtain bounded growth curves and insofar it differed slightly from the conventional approach of adding small random numbers.) These new parameters were accepted as new candidates, if for them *SSE* became smaller, but in order to escape from suboptimal solutions, with a certain probability new parameter values were also accepted, if for them *SSE* became larger. (Otherwise, the old candidates were retained.) This step was then repeated with the accepted parameter. At the begin of each inner loop, i.e. at a grid point near the diagonal of the form (*a*, *a* + 0.01), the optimization of *p*, *q*, *v*_0_ used 50,000 of these simulated annealing steps, starting with the estimate *p* = *q* = 1 and the initial condition *v*(*t*_1_) = *v*_1_ (first data point). For the subsequent grid points in the *b*-direction (inner loop), the previous optimization results were used as starting values and improved in 10,000 annealing steps. Thereby, after each 1000 steps the simulated annealing restarted with the hitherto best parameters. Further, the probability of accepting parameters with a higher *SSE* was lowered slightly. (This was made dependent on the hitherto obtained optimization results and is known as adaptive cooling: [43].) To assess the output, a plot of the near-optimal exponent-pairs with threshold 1% for near-optimality (i.e. *SSE*_*opt*_ exceeded the minimum *SSE* by at most 1%) was visually inspected. Where the plot had a frayed appearance, the optimization exercise was repeated with more simulated annealing steps. CPU-time for the computations took about one week.

The optimal parameters from simulated annealing were finally used as starting values for a nonlinear regression for the model with the optimal exponent-pair, using standard methods to determine *v*_0_, *p*, *q* (Mathematica command NonlinearModelFit) and further improve *SSE*_*opt*_.

## Results

### Growth over 114 days

**Fig. 3:**
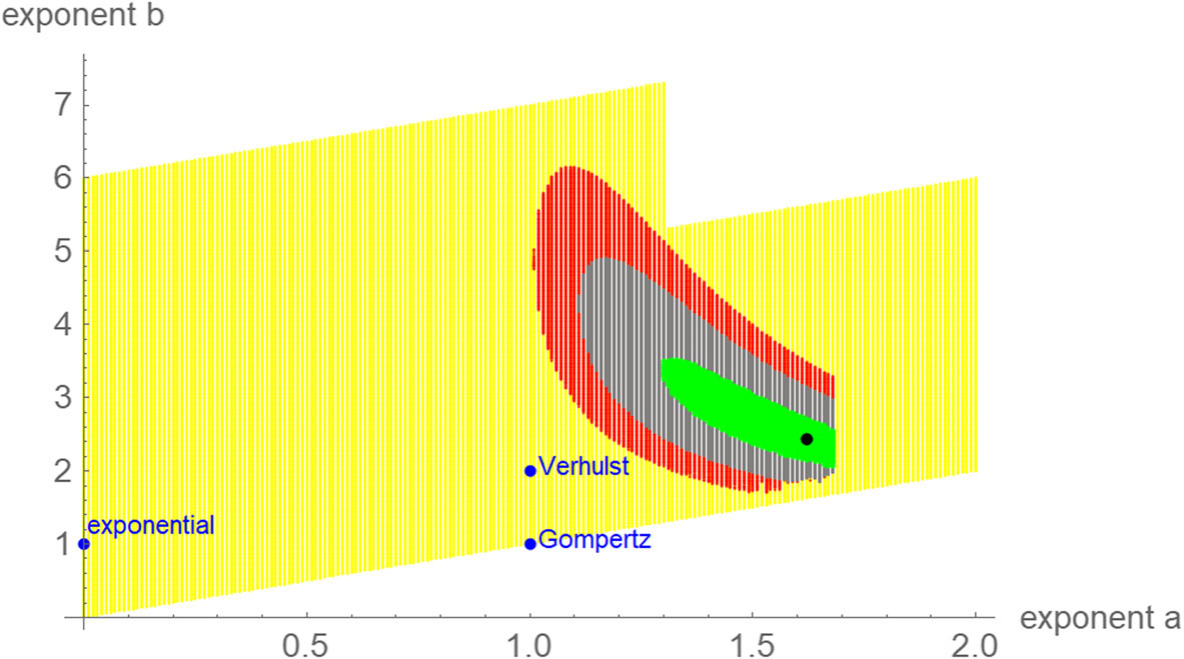
Extended search grid (yellow) with 106,599 grid-points; selected exponent-pairs (blue); optimal exponent-pair (black) *a* = 1.62, *b* = 2.44 for the fit to the growth data over 114 days; 17,403 and 9,416 and 2,315 near-optimal exponent-pairs (red, gray, and green) for the thresholds 10, 5, and 1%, respectively (i.e. for the exponent-pairs *SSE*_*opt*_ exceeded the minimal *SSE* by at most that threshold). The optimal parameters obtained from simulated annealing are displayed in Table 2. The parameters were slightly improved in Fig. 4

**Fig. 4:**
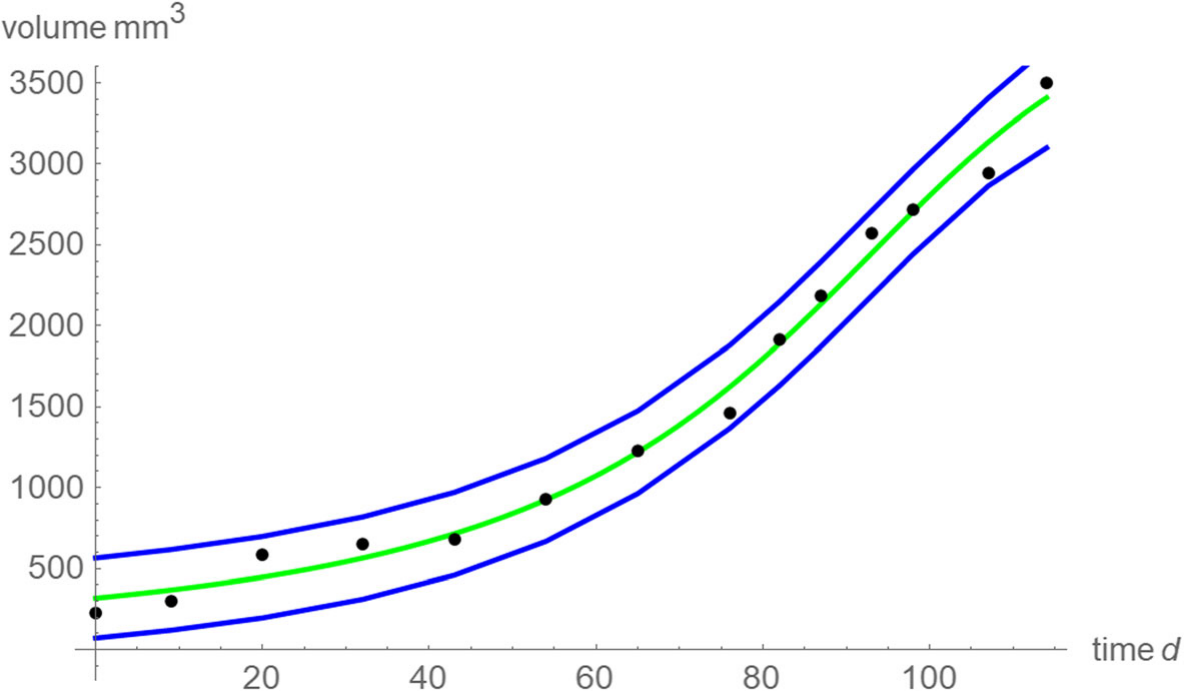
Data (black dots); single prediction band (95% confidence: blue); best fitting model curve (green): optimal exponent-pair *a* = 1.62, *b* = 2.44 and (slightly improved) parameters *v*_0_ = 317.9 mm^3^ (95%-confidence limits, 249.2 to 386.5), *p* = 5·10^− 4^ (4·10^− 4^ to 6.1·10^− 4^) and *q* = 5.6·10^− 7^ (3.7·10^− 7^ to 7.4·10^− 7^)

The best fitting model satisfying eq. (1) was sought for. Figure 3 plots the extended search grid, the optimal and the near-optimal exponent-pairs for different thresholds. Simulated annealing achieved the best fit *SSE*_*opt*_ (1.62, 2.44) = 1.274·10^5^ with the parameters *v*_0_, *p*, and *q* from the caption of Fig. 3. Another round of simulated annealing (50,000 steps to optimize also the exponent-pair) did not improve the solution. However, using the parameters *v*_0_, *p*, and *q* as starting values for a standard nonlinear regression for the model defined from the exponent-pair *a* = 1.62, *b* = 2.44 resulted in a slight improvement below the displayed accuracy. Figure 4 plots the best fitting model curve. For comparison, amongst the seven models of [31] the best fit was achieved for the logistic model (Verhulst) with *SSE*_*opt*_ = 1.61·10^5^. ([31] obtained the slightly worse value *SSE*_*opt*_ = 1.67·10^5^: Fig. 3 of the cited paper.) Thus, *SSE*_*opt*_ of the Verhulst model exceeded the least *SSE* by 26%.

The best fitting model curve supported the hypothesis of bounded growth, as its asymptotic volume of 4,034 mm^3^ (computed as the limit of the model curve *v*(*t*) for infinite *t*) remained close to the maximally observed volume (16% increase from 3,503 mm^3^, whereas 50% increase might be excessive [32]) and as the inflection point could be discerned from the data. (It was attained during the observed time span at the volume of 2,450 mm^3^, which is 70% of the maximally observed volume.) Further, as shown by Fig. 4, the best fitting model curve was close to the data whence there did not arise concerns about outliers in the data or about the convergence of optimization; the standard deviation of the fit residuals was 99 mm^3^. Distribution fit tests did not refute the implicit assumption for using the method of least squares, normally distributed fit residuals (*p*-value 0.42 for a sign test for median 0 and *p*-values 0.66–0.67 for the Anderson-Darling and Cramér-von Mises tests for normality).

### Predictive power

To explore the potential for prognosis, [31] fitted several models to the first seven growth data covering a time span of 65 days. This paper therefore repeated the above computations for the data of the first 65, 76, 87, 98, and 107 days and compared them with the full data.

**Fig. 5:**
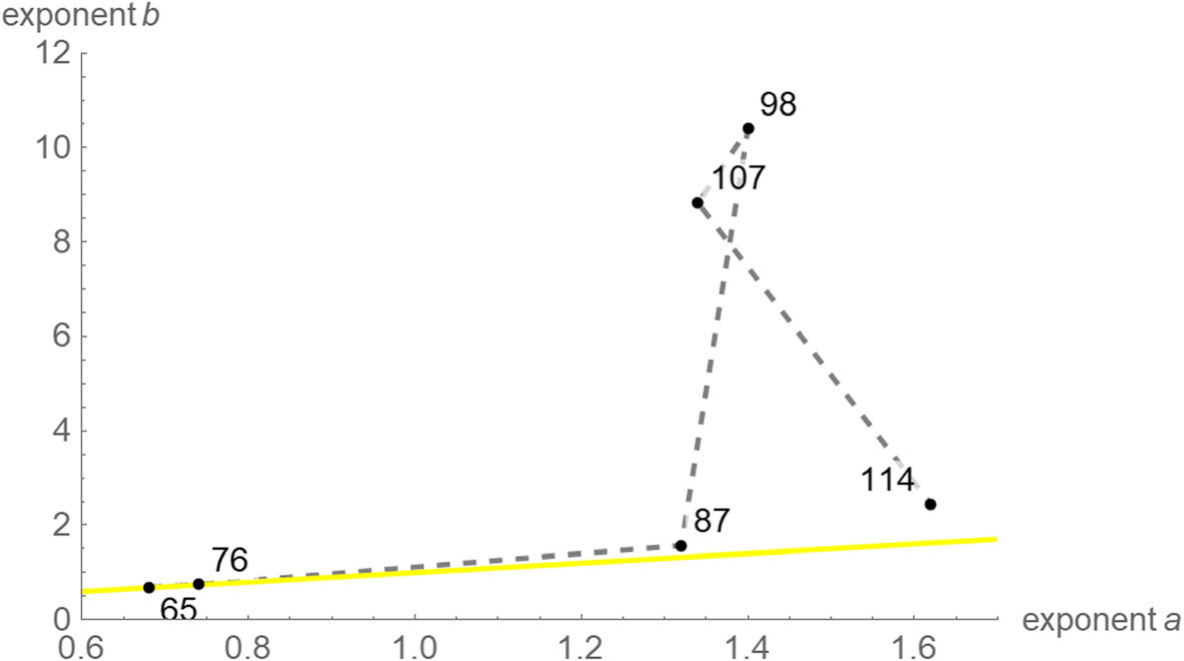
Optimal exponent-pairs for different data, labeled by their time spans of observation. The yellow line is the lower bound for the exponent-pair region (diagonal *a* = *b*)

Table 2 reports the optimal exponent-pairs and parameters of the best fitting model curves for each of these data and Fig. 5 plots the optimal exponent-pairs (labeled by the considered time spans). For the data over a time span of 65 days, [31] identified the von Bertalanffy model as best fitting model and reported *SSE* = 33,700 (caption to Fig. 1 of that paper). Simulated annealing improved this fit for the von Bertalanffy model to *SSE*_*opt*_ (0.67, 1) = 32,177 and identified a still smaller *SSE*_*opt*_ (0.68, 0.69) = 32,087 (rounding to integers).

**Table 2:** Optimal exponents and parameters for different data

Time span	Exponents		Parameters			Goodness of fit	
	*a*	*b*	*v*_0_	*p*	*q*	*SSE*_*opt*_	*SSE* (all)
65	0.68	0.69	266.2	0.185	8.66·10^−3^	3.21·10^4^	2.37·10^6^
76	0.74	0.75	270.3	0.140	0.019	3.22·10^4^	1.83·10^6^
87	1.32	1.57	321.5	2.66·10^−3^	1.47·10^− 5^	5.57·10^4^	5.69·10^6^
98	1.4	10.4	331.7	1.50·10^−3^	1.36·10^− 34^	5.83·10^4^	5.55·10^5^
107	1.34	8.83	323.5	2.27·10^−3^	2.28·10^−29^	6.12·10^4^	3.63·10^5^
114	1.62	2.44	318.5	5.02·10^−4^	5.55·10^−7^	1.274·10^5^	

Note: The table reports the results of simulated annealing (rounded) without additional optimization steps. The first column identifies the data by their time spans (days of observation). As to the last columns, *SSE*_*opt*_ was computed over the indicated timespan and *SSE* (all) was computed over the data for 114 days

**Fig. 6:**
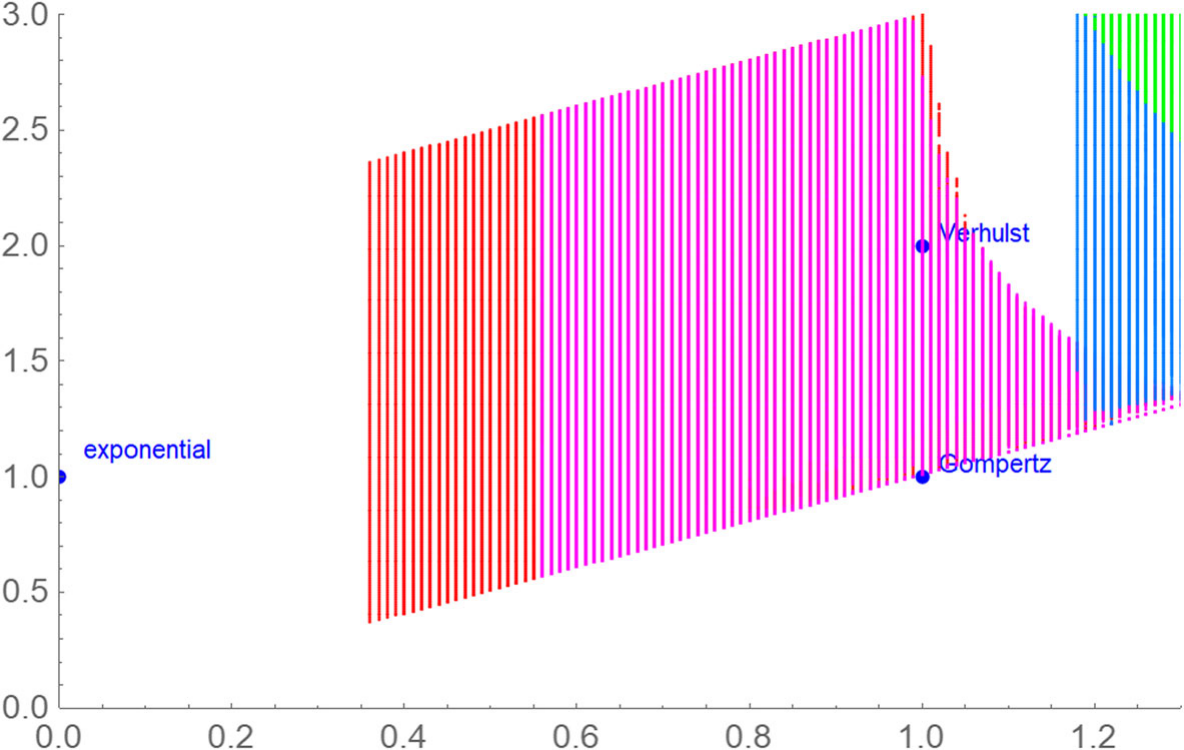
Regions of near-optimal exponent-pairs within the search grid of Fig. 1 for four data, whose *SSE*_*opt*_ did not exceed the minimal *SSE* for the respective data by more than 5%: data for 65 days (red, violet and the lower part of blue); for 76 days (violet and the lower part of blue); for 87 days (blue and green); and for 114 days (green). The regions for 98 and 107 days were outside the considered search grid. The exponent-pairs of three named models were displayed for better orientation (dark blue)

Figure 6 is the counterpart to Fig. 3 but restricted to near-optimal exponent-pairs within the initial search grid of Fig. 1 and using the 5% threshold for defining near-optimality. (This threshold reduced overlaps.) Except for the data over 65 and 76 days, all optimizations needed extensions of the initial search grid of Fig. 1. Compared to Fig. 3 (gray region) the region of near-optimal exponents for the data over a time span of 65 days was huge. This high variability indicates that the data did not suffice to identify a suitable growth model. One reason was the small number of only seven points of time for fitting a solution of eq. (1) with five free parameters. This was demonstrated by the region of near-optimal exponent-pairs for the data over a time span of 76 days, which was smaller.

**Fig. 7:**
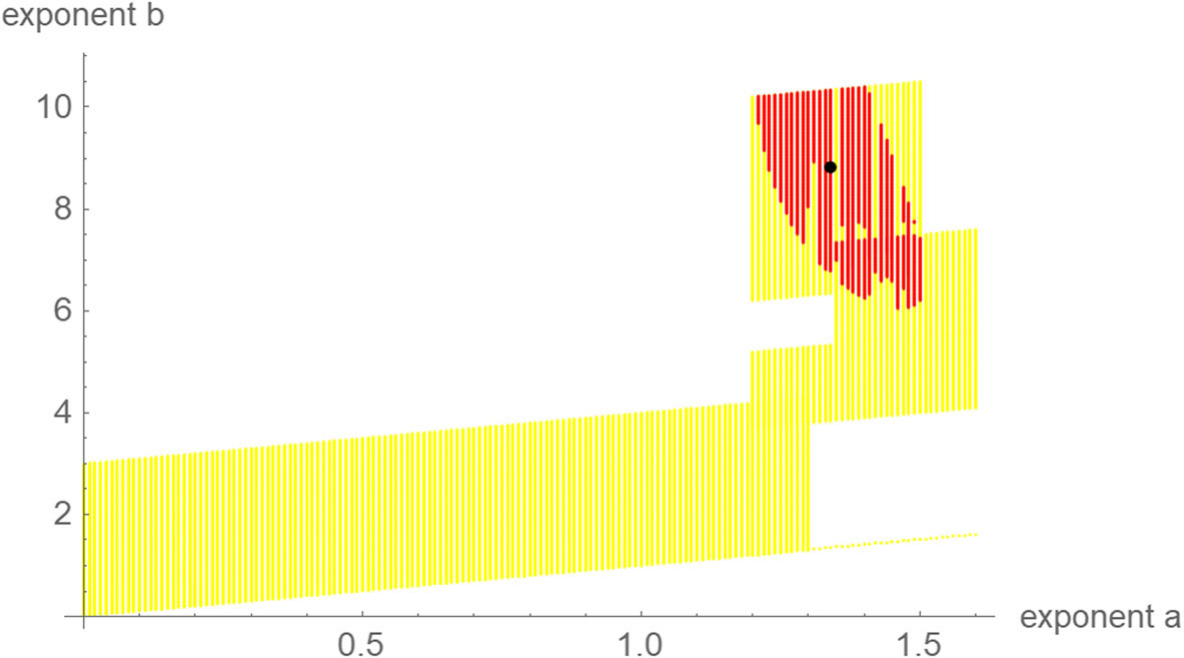
Search grid (yellow), optimal exponent-pair (black) for finding the best fitting model curve to the data of the first 107 days of tumor growth, and near-optimal exponent pairs (red), using a threshold of 5%

The optimization for the data for 98 and 107 days was particularly time consuming, as 63,377 and 64,150 grid points were searched. For the latter data, Fig. 7 plots the search grid (its zig-zag shape was due to the successive addition of grid points) and the optimal (black) and near-optimal (red, threshold 5%) exponent-pairs. For these models, the large exponents, *b*, necessitated the use of extremely small parameters, *q*. The frayed character of the red region reflects the numerical problems of using such exponents and parameters; due to such problems conventional all-purpose optimization software was doomed to fail. For the former data, the optimal exponent-pair was still on the upper boundary of the search grid, whence the optimality of the exponent-pair was not secured.

**Fig. 8:**
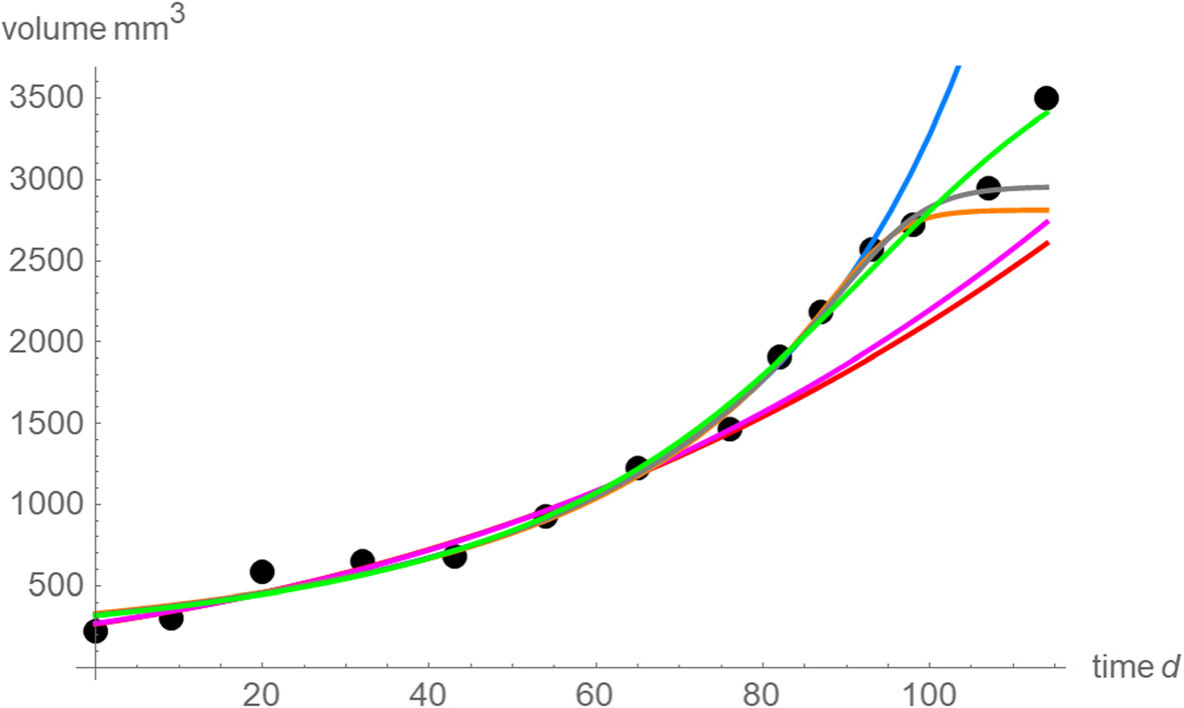
Model curves (exponents and parameters in Table 2) with the best fit to the following data (black dots): data for 65 days (red); data for 76 days (violet); data for 87 days (blue); data for 98 days (orange), data for 107 days (gray) and data for 114 days (green)

Figure 8 plots the optimal model curves defined in Table 2. Each model curve had a good fit to the data that it intended to approximate. For most curves the fit to the next data point was acceptable, but the prognosis for more than 10 days was poor.

## Discussion

Our results confirm the finding of [31], that the selection of the model with the best fit to an initial segment of the data may “not guarantee the selection of the best model for predicting future behavior”, which we represented by the full dataset. However, our conclusion differs: The failure of prognosis may not necessarily be due to the choice of a false model. Rather it may be the data that limit the time horizon for forecasting.

Figure 8 explains the reasons for the failure of the prognosis for the present data. The red curve was fitted to the first seven data (65 days) and its prognosis for day 76 was acceptable, as it extrapolated the apparent trend, whereas its prognosis for the remaining days was too low. The violet curve (76 days) extrapolated this trend, too, and so its prognosis failed. The blue curve was fitted to the first ten data (87 days) and it correctly identified another trend with a steeper ascent till day 93. However, its extrapolation for the following days was too high. The orange and the gray curves used the first 12 and 13 data points (98 and 107 days) and they identified the slowing down of growth, but they overestimated it and could not forecast the volume for the last data point (day 114). Thus, the present data seemed to display two apparent changes of trend, an acceleration of growth after day 76 and a slowing down after day 93, resulting in the typical S-shape of bounded growth.

For a practitioner, who uses the past data to extrapolate into the future, the failure of forecasting may indicate problems for the patient, e.g. a different phase of growth, where the apparent trend of the growth curve changes due to a biological cause (e.g. angiogenesis). It may indicate problems with the data, such as the presence of outliers. Or it may merely indicate that the true nature of the growth curve could not be identified, because its S-shape could not (yet) be discerned from the data.

**Fig. 9:**
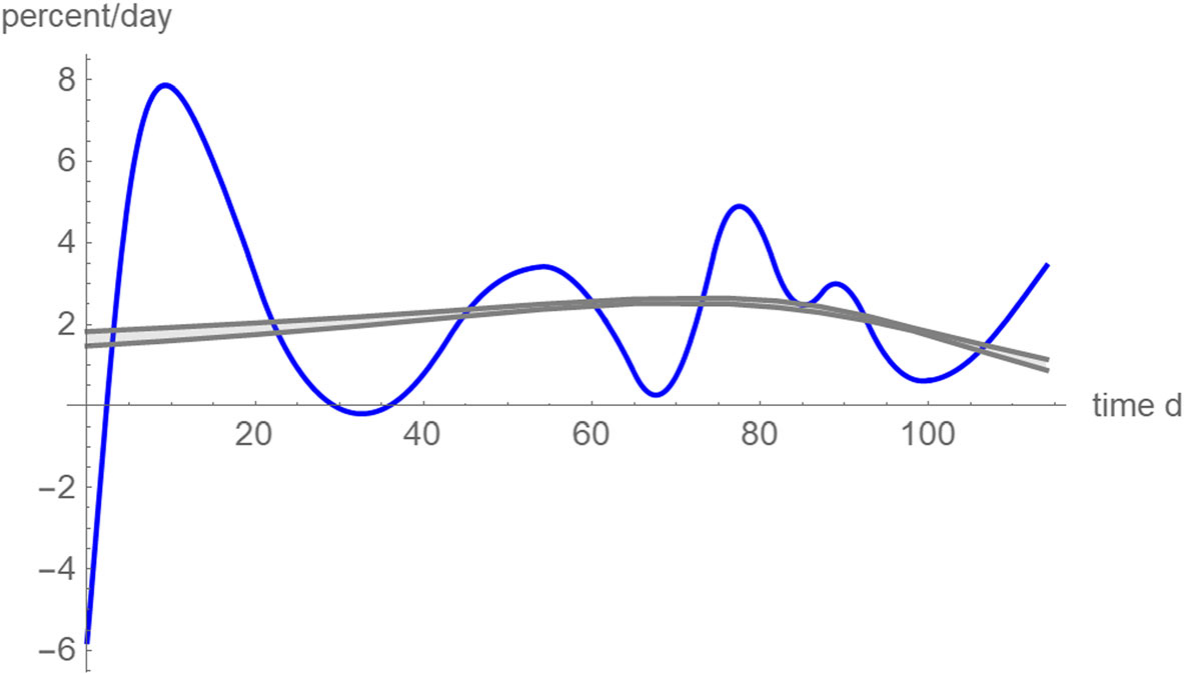
Relative growth rates (percent/day) of the best fitting model curves from 2,315 near-optimal exponent-pairs (their *SSE*_*opt*_ exceeds the minimal *SSE* by at most 1%). The shaded area is the region between the minimal and maximal growth rates that some model reached at that day. The blue curve is the relative growth rate computed from the spline interpolation function of Fig. 2 (a method for the numeric differentiation of the data)

For the present data the latter reason may apply, as Fig. 8 displays a growth curve with a good fit to the data (green curve) and Fig. 4 shows that with 95% confidence all observations were within its single prediction band (no outliers). Figures 9 confirms this. It uses the data for all 114 days of observation and plots the relative growth rates *v´*/*v* over time for the best fitting models of the top-1% of the near-optimal exponents. Its reverted U-shape suggests that the tumor size may have approached the carrying capacity, whence further growth would be inhibited by lack of resources, unless other drivers of growth (e.g. angiogenesis) were activated. This information might not have been readily available, if *v´*/*v* were estimated from a numeric differentiation of the data (blue line).

**Fig. 10:**
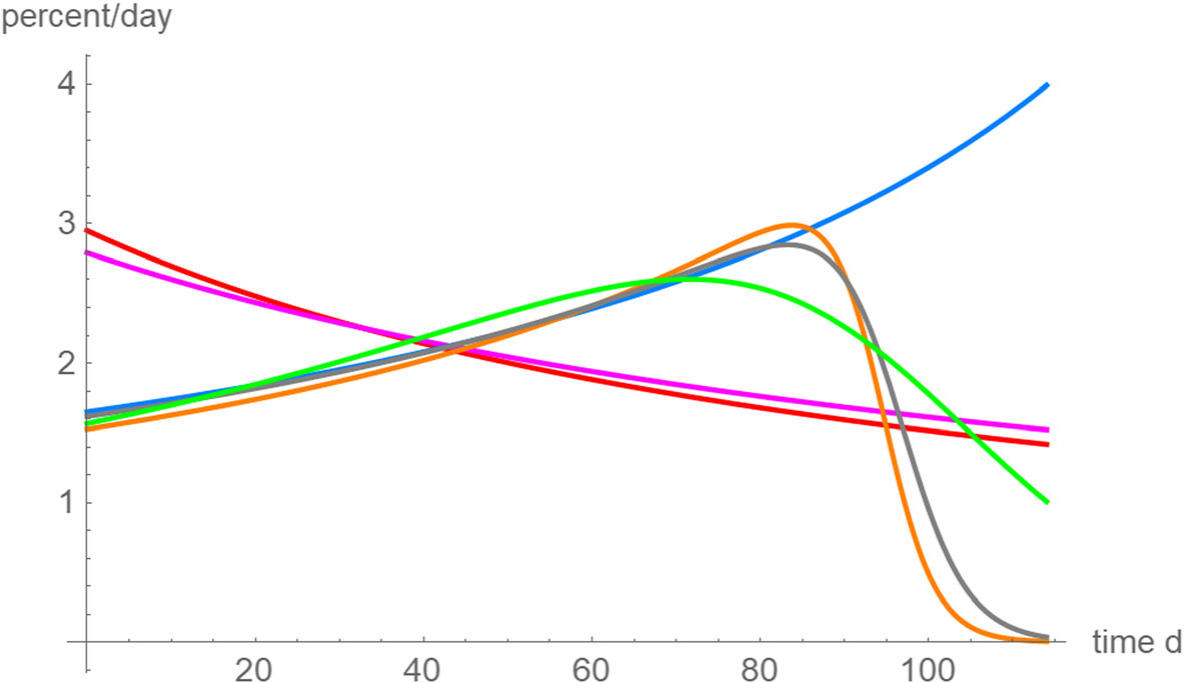
Relative growth rates (percent/day) based on the best fitting model curves for different data: data for 65 days (red); data for 76 days (violet); data for 87 days (blue); data for 98 days (orange), data for 107 days (gray) and data for 114 days (green)

The analysis of relative growth rates in Fig. 10 confirms the conclusion that the different forecasts may have been due to apparently different trends, that nevertheless could be reconciled into one well-fitting model function. Judging only from the initial data till day 76 the relative growth rate appeared to slow down. With the data for 87 and more days, this picture changed; the best fitting model curves had increasing relative growth rates also for the initial days. However, the data for the first 87 days could not recognize the subsequent slowing of growth. Thereby, owing to the lack of more long-term observations, the models based on the data for 98 and 107 days overestimated this slowing.

Further, the size of the region of near-optimal exponent-pairs is related to the information inherent to the growth data: The larger the region is, the fewer information can be retrieved, as for a larger region the data would be compatible with more (too many) possible shapes of the growth curve. As was shown in Fig. 6, the data for 65 days resulted in a huge region, whence no reliable prognosis could be expected. For the full set of data for 114 days, the region of near-optimal exponents was smaller (Fig. 3).

## Conclusions

For the data of [31] the prognosis of tumor growth was feasible only for a short time span into the future: Past growth data could not identify, if and when there would be a change in the apparent trend or even a change in the biological mechanism of growth. Insofar, the data appeared to be peculiar, but we did not check, if this peculiarity would be typical for growth data of cancer. For instance, concerning biological interpretations of the best fitting model curve, the exponent-pairs of the named models were remote from the optimal and near-optimal exponent-pairs for the data over 114 days (Fig. 3). Further, the optimal exponent-pairs obtained from initial segments of the data did not show a clear pattern (e.g. convergence) that would relate them to the optimal exponent-pair of the data over 114 days (Fig. 5). Thus, the biophysical arguments that supported the named models may not apply in the present context.

However, even for peculiar data, prognosis is not futile, as for practitioners any discrepancy between observed and forecasted growth may be an important warning signal that the tumor biology may change. The present paper provided methods for a more accurate prognosis.

In addition to prognosis, practitioners may use best fitting model curves to assess the character of past growth in terms of the relative growth rate *v´*/*v*. However, for the present data also this analysis of the past did depend on how much information about the growth was available at the time when the assessment was done. For, the assessment switched from an initially decreasing relative growth rate, if only seven or eight data points were considered, to an initially increasing relative growth rate, when more data were utilized (Fig. 10).

## Data Availability

The method explains the sources of the data. Further, the authors provided supplementary material, namly an spreadsheet (MS Excel) with the optimization results for the full data set and the Mathematica file that produced this Excel file.

## Abbreviations

SSE: is the sum of squared errors (i.e. the fit residuals)
